# Physicochemical Properties and Biological Activities of Garden Cress (*Lepidium sativum* L.) Seed and Leaf Oil Extracts

**DOI:** 10.1155/2022/2947836

**Published:** 2022-03-16

**Authors:** Fikremariam Adera, Zekeria Yusuf, Mulugeta Desta

**Affiliations:** School of Biological Sciences and Biotechnology, Haramaya University, Dire Dawa, Ethiopia

## Abstract

Garden cress (*Lepidium sativum* L.) seed is a rich source of proteins, dietary fiber, omega-3 fatty acids, iron, and other essential nutrients and phytochemicals. The aim of the present study was to investigate the physicochemical properties and biological activities of garden cress (*L. sativum*) seed and leaf oil extracts using solvent extraction methods. The result indicated that oil yield (25.75 ± 2.48%) and specific gravity (0.84 ± 0.08) were significantly (*P* < 0.05 based on least significance difference *t*-test) higher for seed oil extract. Seed oil also presented significantly (*P* < 0.05) higher antioxidant activities with respect to ascorbic acid content (24.21 ± 3.04%) and DPPH (2, 2-diphenyl-1-picrylhydrazyl) (26.55 ± 0.21%) values. The leaf oil extract has exhibited stronger antibacterial activity with a maximum zone of inhibition (18.50 mm), a minimum inhibitory concentration (MIC) of 0.05 *µ*g/ml, and a minimum bactericidal concentration (MBC) of 0.05 *µ*g/ml against *Staphylococcus aureus*. Leaf oil extract has also demonstrated stronger antifungal activity with a maximum zone of inhibition (18.50 mm), MIC (0.25 *µ*g/ml), and a minimum fungicidal concentration (MFC) of 0.50 *µ*g/ml against *Aspergillus Niger*. The result suggesting that leaf oil presented superior antimicrobial but inferior antioxidant potential than seed oil in garden cress.

## 1. Introduction

*Lepidium sativum* belongs to *Cruciferae* (Brassicaceae) family [1, 2]. Its common name is garden cress and it is locally called Shifu in Oromo, and it is believed to have originated in Ethiopia [3]. All parts of garden cress including seeds, leaves, and roots possess economic importance [1]. However, the plant is cultivated mainly for seed [4]. The garden cress seed is galactogogue, bitter, thermogenic, depurative, rubefacient, aphrodisiac, ophthalmic, antiscorbutic, antihistaminic, diuretic, and acts as a tonic [5]. It is used as a remedy of various diseases such as asthma, coughs with expectoration, diarrhea, dysentery, poultices for sprains, leprosy, skin disease, splenomegaly, dyspepsia, lumbago, leucorrhoea, scurvy, and seminal weakness, which can be treated using garden cress seed [6]. The garden cress seed is composed of 80–85% endosperm, 12–17% seed coat, and 2–3% embryo; 25% protein, 14–24% lipids, 33–54% carbohydrates, and 8% crude fiber [7]. Garden cress seed oil is rich in healthy fatty acids and natural antioxidants such as vitamin A and E and eugenol which help to protect cells from damage by free radicals [2].

For the compensation of proteins and fat components, garden cress extract or powder can be used as food additives [2]. Since garden cress also acts as a thickening agent, the combination of both juices and extract may lead to the formation of health-promoting beverages having good textural, sensory attributes, and nutritional properties [2]. A beverage was developed by combining lime juice and saccharin, honey, and garlic for the compensation of proteins and fat [8]. Fresh leaves of garden cress are mainly used in salads and have antibacterial, diuretic, and stimulant properties. The juice of an eight-day-old whole garden cress plant has been shown to be chemoprotective against IQ (2-amino-3-methylimidazo quinoline)-induced genotoxic effects and colonic neoplastic lesions in rats [9].

Garden cress seeds have been shown to reduce the symptoms of asthma and improve lung function in asthmatics [10]. The seeds have been reported as possessing hypoglycemic properties, and the seed mucilage is used as a substitute for gum Arabic [11]. In Ethiopia, even though the plant is used in various food preparations, spice, and traditional medicinal properties, little research work has been carried out on the garden cress varieties cultivated in Ethiopia. Therefore, the aim of this study was to investigate physicochemical properties and biological activities (as antioxidant and antimicrobial activities) of the oil extracts from garden cress (*L. sativum*) seeds and leaves using solvent extraction method.

## 2. Materials and Methods

### 2.1. Garden Cress Sample Collection and Preparation

**Figure 1:**
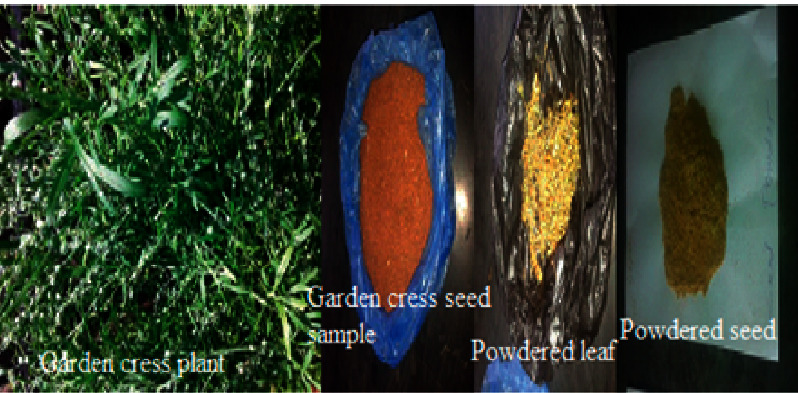
Sample garden cress plant.

The study was conducted in the Biotechnology Laboratory, Haramaya University. The garden cress (*Lepidium sativum* L.) plant samples (Figure 1) were collected from the Kosober district, West Gojam, Ethiopia. The seeds and leaf samples were manually washed with distilled water and residual moisture was evaporated at room temperature. Then, after grinding to a fine powder in a grinder for 2 min, the process was stopped for 15 sec to avoid heating of the samples. The determination of moisture (on a dry basis) was carried out as per the standard method [12].

### 2.2. Oil Extraction and Determination of Physicochemical Properties and Antioxidant Activities

The oil extraction was carried out in a Soxhlet apparatus (Bionics Scientific) using petroleum ether (Sigma-Aldrich) as a solvent. Eighty grams (80 gm) of powdered garden cress leaf and seed samples were dissolved in a 480 ml petroleum ether solution and kept in the Soxhlet apparatus for 8 hours. The crude oil extracts were concentrated in a water bath by adding sodium sulfate. The determination of physicochemical properties including specific gravity, oil yield, acid value, free fatty acid value, and peroxide value. The antioxidant activities were determined based on ascorbic acid content, DPPH, and hydrogen peroxide free radical scavenging activities.

The oil yield and specific gravity were determined as per the standard method of Association of Analytical Chemists [12]. The % oil yield of each sample was determined as follows:

(1) $$\begin{array}{c} \text{oil yield}=\frac{\text{oil weight}\left(\text{OW}\right)}{\text{sample weight}\left(\text{SW}\right)}\times 100, \end{array}$$

where oil weight = W2–W1, W1 = weight of the extraction flask (g); W2 = weight of the extraction flask plus the dried crude fat (g).

The specific gravity of the oil was determined gravimetrically by employing the weight ratio of the oil to the equivalent amount of water according to the following formula:

(2) $$\begin{array}{c} \text{specific gravity}=\frac{W2}{W1}, \end{array}$$

where W2 and W1 are the weights of oil and equivalent amount of water, respectively.

### 2.3. Determination of Acid Value

The acid value was determined using the standard method [12]. In brief, 2 g of oil sample was weighed into a 250 ml conical flask and then 25 ml of diethyl ether mixed with 25 ml of alcohol and 1 ml of 1% phenolphthalein indicator were added to the oil sample. The conical flask was then placed in a hot water bath until the oil was completely dissolved in the solvent. The hot solution was then titrated with 0.1 M KOH (Sigma-Aldrich) until a pink color which persisted for 15 seconds was noticed. The acid value was calculated as follows:

(3) $$\begin{array}{c} \text{acid value}=\frac{\text{titre}\left(\text{ml}\right)\times \;5.61}{\text{weight of sample used}}. \end{array}$$

Acid value expressed as acid value (mg KOH/g of oil).

### 2.4. Estimation of Free Fatty Acid

The percentage of free fatty acid (% FFA) was estimated by multiplying the acid value with the factor 0.503. The %FFA = 0.503 × acid value.

### 2.5. Determination of Peroxide Value

To a weighed sample (1.0 g) in a flask, powdered potassium iodide (1.0 g) and solvent mixture (2 : 1, glacial acetic acid: chloroform v/v) were added. The resulting solution was then placed in a water bath to dissolve properly, and 5% potassium iodide (20 cm^3^) was added. The sample solution was then titrated with 0.002 N sodium thiosulphate (Sigma-Aldrich) using starch as an indicator. The peroxide value of the samples was calculated using the following equation [13]:

(4) $$\begin{array}{c} \text{PV}=2\times V, \end{array}$$

where PV = peroxide value, *V* = volume of sodium thiosulphate used, 2 = (*N* × 1000)/*W*, *N* = normality of sodium thiosulphate used, and W = weight of sample used.

### 2.6. Antioxidant Activity Tests

#### 2.6.1. DPPH Radical Scavenging Activity

The radical scavenging activity (RSA) of the oil extracts was adopted to measure antioxidant activity using the DPPH method [14]. In brief, 2 mL of the oil extract was added to 2 mL of DPPH (0.1 mM) solution (Suvchem). The mixtures were kept aside in a dark area for 30 min and absorbance was measured at 517 nm against an equal amount of DPPH and methanol as a blank.

The percentage of DPPH inhibition of free radicals was estimated using the following equation:

(5) $$\begin{array}{c} \%I=\left[1-\left(\frac{A_{517\left(\text{sample}\right)}}{A_{517\left(\text{blank}\right)}}\right)\right]\times 100. \end{array}$$

#### 2.6.2. Hydrogen Peroxide Scavenging Activity

The radical scavenging activity of individual extracts was determined using the H2O2 method [15]. In brief, 2 mL of the oil extract solution was added to 4.0 mL of H2O2 (20 mM) solution (Arkema) in phosphate buffer (pH 7.4). After 10 min, the absorbance was measured at 230 nm against the phosphate buffer blank solution. The percentage scavenging of H2O2 was calculated using the following equation:

(6) $$\begin{array}{c} \%\text{scavenging of H}2\text{O}2=\left[\frac{A0-A1}{A0}\right]\times 100, \end{array}$$

where *A*0 = absorbance of the control (phosphate buffer with H2O2) and *A*1 = absorbance of the test extracts.

### 2.7. Determination of Ascorbic Acid

The ascorbic acid content was determined using the 2, 6- dichlorophenol indophenol (DCPIP) dye (Sigma-Aldrich) method [15]. Accordingly, 5 ml of the standard ascorbic acid solution was pipetted into a 100 ml conical flask, and then 5 ml of the 3% HPO_3_ solution was added. The ascorbic acid solution was titrated with the dye solution to a pink color that persisted for 15 sec. The titre value was recorded. The dye factor was expressed as mg of ascorbic acid per ml of the dye. Since 5 ml of the standard ascorbic acid solution contains 0.5 mg of ascorbic acid, the dye factor is as follows:

(7) $$\begin{array}{c} \text{dye factor}\left(\text{mg ascorbic acid per dye}\right)=\frac{0.5\text{ mg}}{\text{titrant volume}}. \end{array}$$

One ml of the extracted oil was diluted to 5 ml with 3% metaphosphoric acid (India Mart) in a 50 ml volumetric flask. The aliquot was then centrifuged (Model, Z300, 580W, 3052 Nm, German) for 15 minutes and titrated with the standard dye to a pink end point (persisting for 15 seconds). The ascorbic acid content was calculated from the titration value, dye factor, dilution, and volume of the sample as follows:

(8) $$\begin{array}{c} \%\text{A.A}=\frac{\left(\text{ABRsample}\right)\times \text{ dye factor}\times \text{volume of initial test solution}}{\text{volume of test solution titrated}}\times 100\%, \end{array}$$

where A.A = ascorbic acid; ABR = average burette reading.

### 2.8. Antimicrobial Activity of the Oil Extracts

The antimicrobial experiment was arranged as 2 × 1 × 4 (two source extracts: seeds and leaves of garden cress at three concentration levels; 1 solvent system, i.e., petroleum ether; and 4 test pathogens including two bacteria: *Escherichia coli* (Gram-negative), *Staphylococcus aureus* (Gram-positive), and two fungal spp.: *Aspergillus Niger* and *Candida albicans*) in a completely randomized factorial design in three replications. The test pathogens were obtained from the Ethiopian Institute of Food and Health, Addis Ababa, Ethiopia. The fungal and bacterial pathogens were subcultured and maintained on potato dextrose agar (PDA) (NEOGEN) and nutrient agar, respectively. Then, the fungal and bacterial cultures were incubated for 72 h at 27°C and for 18–24 h at 37°C, respectively.

#### 2.8.1. Media Preparation and Standardization of Inoculums

Media were prepared and sterilized using an autoclave according to the manufacturers' instructions. Two to three bacterial colonies on the plate were picked up with a sterile inoculating loop and transferred into a test tube containing sterile normal saline and vortexed thoroughly. The spores of the test fungi were harvested by washing the surface of the fungal colony using 5 mL of sterile saline solution (JPAC). This procedure was repeated until the turbidity of each bacterial and fungal spore suspension matched the turbidity of 0.5 McFarland Standards as described by the Clinical Laboratory Standards Institute [16]. The resulting suspension was used as inoculums for the test pathogen in the antimicrobial susceptibility test using the disc diffusion method [17].

#### 2.8.2. Disk Diffusion Method

The Mueller–Hinton agar (MHA) (NEOGEN) plates were inoculated by streaking using the cotton swab three times over the entire surface and rotating the MHA plates approximately 60° each time to ensure an even distribution of the inoculums. Then, the MHA plates were left open for three to five minutes to allow for any excess surface moisture to be absorbed [16]. The impregnated discs were dispensed onto the surface of the inoculated agar plates using sterile forceps. Discs of commercial ciprofloxacin (1 *µ*g/disc) and fluconazole (1 *µ*g/disc) were used as positive controls for bacterial and fungal pathogens, respectively, and the pure solvent (petroleum) impregnated discs were used as negative controls. Then, the MHA plates were sealed with parafilm and incubated at 37°C for 24 hrs and 27°C for 72 hrs for bacterial and fungal pathogens, respectively. Then, the diameters of the zone of inhibition around each disc were measured to the nearest millimeter along two axes (i.e., 90° to each other) and the means of the two readings were recorded.

#### 2.8.3. Determination of Minimum Inhibitory Concentration (MIC)

The oil extracts that showed significant antimicrobial activity in the antimicrobial activity tests were selected for determination of MIC based on the method used by Morshed et al. [18] with slight modifications. The MICs of the oil extracts were determined by the broth dilution method. In the broth dilution method, the oil extract solution, for example, at 1 *µ*g/ml (w/v), was serially diluted in a two-fold dilution as 1 *µ*g/ml, 0.50 *µ*g/ml, and 0.25 *µ*g/ml, 0.125 *µ*g/ml, and 0.0625 *µ*g/ml concentrations. Two milliliter of nutrient broth and potato dextrose broth for bacteria and fungi, respectively, were added into all test tubes, and 0.1 ml of the prepared concentration of each oil extract was mixed with the nutrient broth and potato dextrose. Thereafter, standardized inoculums of 0.1 ml of the respective test pathogens were dispensed into the test tubes containing the suspensions of the broth and the oil extract. Then, all test tubes were properly corked and incubated at 37°C for 24 hrs for bacteria and 27°C for 72 hrs for fungi. After that, they were observed for absence or presence of visible growth. The lowest concentration at which no visible growth of organisms occurred was regarded as the MIC.

#### 2.8.4. Determination of Minimum Bactericidal (MBC) and Fungicidal Concentrations (MFCs)

For the determination of the MBC and MFC, fresh nutrient agar and potato dextrose agar plates were inoculated with one loop full of culture taken from each of the broth cultures that showed no growth in the MIC tubes. That is, MBC/MFC values were determined by subculturing from respective MIC values if, for example, MIC = 0.50 *µ*g/ml (v/v) subculturing was performed as 0.50 *µ*g/ml, 1.00 *µ*g/ml, 1.50 *µ*g/ml, and 2.00 *µ*g/ml up to four acceptable concentration levels. Since antibacterial agents are usually regarded as bactericidal if the MBC is no more than four times the MIC [16]. MBC/MFC is the amount of the extract that kills microbial growth. While MBC assay plates were incubated for 48 h, MFC assay plates were incubated for 3 days. After the incubation periods, the lowest concentration of the extract that did not allow any bacterial or fungal growth on solid medium was regarded as MBC and MFC for the extract [17]. This observation was matched with the MIC test tube that did not show evidence of growth after 48 h of incubation for bacteria or spore germination after 3 days of incubation for fungi.

The experimental data (Tables 1, 2, and 3 data file deposited as Supplementary material (available here)) were analyzed using SAS version 9.2 [19] to investigate statistical significance between the different oil quality parameters. Differences between means were considered statistically significant at *P* < 0.05 based on least significance difference (LSD) *t*-test.

## 3. Results

### 3.1. Physicochemical Properties of Garden Cress (*Lepidium sativum* L.) Seed and Leaf Oil Extracts

The physicochemical properties of *L. sativum* seed and leaf oil extracts were determined based on oil yield, specific gravity, acid value, free fatty acids, and peroxide values, as shown in Table 1. Significant differences (*P* < 0.05) between seed and leaf oil extracts were obtained for all measured parameters. It was found that oil yield (25.75 ± 2.48%) and specific gravity (0.84 ± 0.08) were significantly (*P* < 0.05) higher for seed oil extract. The other parameters including acid value (2.95 ± 0.20), free fatty acid value (1.48 ± 0.09%), and peroxide value (7.50 ± 0.71%) were significantly (*P* < 0.05) higher for leaf oil extract.

### 3.2. Antioxidant Activities of Garden Cress Seed and Leaf Oil Extracts

The antioxidant activities (Table 2) of garden cress seed and leaf oil extracts presented significantly (*P* < 0.05 based on LSD) higher antioxidant activities with respect to ascorbic acid content (24.21 ± 3.04%) and DPPH value (26.55 ± 0.21%) for seed oil than for leaf oil. However, significantly (*P* < 0.05 based on LSD) higher hydrogen peroxide free radical scavenging activities HPSA (38.10 ± 0.42%) was obtained for leaf oil extract. The result of the present study indicated reverse activity between DPPH and HPSA.

### 3.3. Antimicrobial Activities of Garden Cress Seed and Leaf Oil Extracts

**Figure 2:**
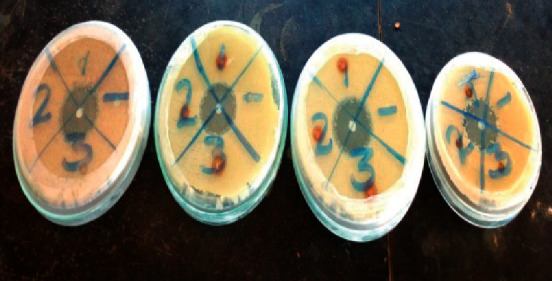
Zone of inhibition diameter showing the antimicrobial activity of the oil extracts.

The diameter of inhibition zone for garden cress seed and leaf oils is shown in Table 3 and Figure 2. Significance (*P* < 0.05 based on LSD) differences were observed for both seed and leaf oil extracts at different concentration levels. The mean zone of inhibition at the highest concentration (3 *µ*g/ml) against bacterial test pathogens ranged from 15.53 ± 0.45 mm to 18.50 ± 0.45 mm, while 12.57 ± 0.55 to 18.50 ± 0.50 mm against fungal test pathogens. Stronger antibacterial activity with maximum zone of inhibition (18.50 mm) was recorded for leaf oil extract against *S. aureus* while the weaker antibacterial activity (15.53 mm) was observed for *L. sativum* seed oil against *E. coli* indicating that *S. aureus* was more susceptible than *E. coli*. Hence leaf oil has exhibited more antibacterial potential than seed oil in garden cress.

On the other hand, the stronger antifungal activity with a maximum zone of inhibition (18.50 mm) was recorded for leaf oil against *A. Niger* as the weaker antifungal activity with a minimum zone of inhibition (12.87 mm) was observed for seed oil against *C. albicans* suggesting leaf oil extract might be more effective antifungal potential than seed oil extract in garden cress.

### 3.4. Minimum Inhibitory Concentration (MIC), Minimum Bactericidal Concentration (MBC), and Minimum Fungicidal Concentration (MFC) of Garden Cress Seed and Leaf Oil Extracts

The effectiveness of *L. sativum* seed and leaf oil extracts against pathogenic microbes was evaluated by MIC, MBC, and MFC, as shown in Table 4. The leaf oil extract has exhibited stronger bactericidal activity with MIC (0.05 *µ*g/ml) and MBC (0.05 *µ*g/ml) against *S. aureus*, while the weakest bactericidal activity with MIC (0.75 *µ*g/ml, the largest value) and MBC (1.25 *µ*g/ml) was recorded for seed oil against *E. coli* indicating that *S. aureus* is more susceptible to the oil extract than *E.coli*, and also indicating that leaf oil extract possesses stronger antibacterial potential than seed oil in garden cress.

By contrast, *L. sativum* leaf oil extract has demonstrated the strongest antifungal activity with MIC (0.25 *µ*g/ml, the least value) and MFC (0.50 *µ*g/ml) against *A. Niger*, whereas the weakest antifungal activity with MIC (1.00 *µ*g/ml) and MFC (1.75 *µ*g/ml) was observed for the seed oil extract against *C. albicans* showing that *A. Niger* was more susceptible to the oil extract than *C. albicans* and the leaf oil was more effective antifungal potential than the leaf oil in *L. sativum*. Alqahtani et al. [23] tested the antimicrobial activity of *L. sativum* oil (LSO) against different bacteria and fungi and clearly presented that all of bacteria and fungi tested were susceptible to LSO, for which the MIC was 47.5 mg/ml, except for *S. enterica*, which showed a higher MIC of 90 mg/ml. The MBC of garden cress oil was found to be equivalent to 100 mg/ml for inhibiting the growth of all bacteria and fungi. This comparable antimicrobial activity against the tested Gram-negative and Gram-positive bacteria and the fungus reveals that LSO exhibits broad-spectrum antimicrobial action.

## 4. Discussion

The acid value, specific gravity, and peroxide values obtained in the present study were lower than those reported in the previous study by Solomon et al. [20] who obtained acid value (4.50), specific gravity (0.9246), and peroxide value (3.42). Since oil quality can be affected by geographical distribution, extraction conditions, and the nature of the plant genotype, the oil extract in the present study demonstrated better oil quality. Since the acid value is lower and the peroxide value shows oil stability. The acid value is the weight of potassium hydroxide in mg required to neutralize the organic acids present in 1 g of the substance.

The acid value is often a good measure of hydrolytic rancidity which has an adverse effect on the quality of many lipids. Specific gravity is a parameter used to measure the volatility of oil extract and confirm the purity of substances. In the present study, the value of specific gravity obtained at 0.49 for leaf oil and 0.84 for seed oil is less than 1, indicating that the oil is less dense than water suggesting that the oil is composed of light molecular weight components and therefore is volatile. For quality oil, the value specific gravity should be close to one. That means less volatile and better purity. Acid value is used as an indication of edibility of oil and suitability to be used in the paint industry and that are within range of 1.26 to 2.95 falls within the recommended codex of 0.6 and 10 for virgin and nonvirgin edible oils and fats [21]. The peroxide value of *L. sativium* seed oil is low (2.20 meqKOH/g) compared to the maximum acceptable value of 10 meqKOH/g set by the Codex Alimentarius Commission for groundnut seed oils [22].

Hydrogen peroxide scavenging activity (HPSA) is an antioxidant parameter used to remove H2O2 which is toxic by product of the cell. The oil extracts in the present study was assessed for their tendency to remove H2O2. The higher DPPH antioxidant activities in seed oil extract indicate the presence of higher essential omega-3 fatty acids in garden cress seed oil extract. The antioxidant activities of seed oil were found to be significantly higher than those of leaf oil extract suggesting that seed oil might possess better biological activities, oil quality, and potential pharmacological applications. A similar study was conducted by Alqahtani et al. [23] who reported the garden cress seed oil antioxidant activity in a dose dependent pattern, with a half maximal inhibitory concentration (IC50) value of 40 mg/ml.

The leaf oil extract has exhibited stronger antibacterial activity with a maximum zone of inhibition (18.50 mm), a minimum inhibitory concentration (MIC) of 0.05 *µ*g/ml, and a minimum bactericidal concentration (MBC) of 0.05 *µ*g/ml against *Staphylococcus aureus*. Leaf oil extract has also demonstrated stronger antifungal activity with a maximum zone of inhibition (18.50 mm), MIC (0.25 *µ*g/ml), and a minimum fungicidal concentration (MFC) of 0.50 *µ*g/ml against *Aspergillus Niger*. The result suggesting that leaf oil presented superior antimicrobial but inferior antioxidant potential than seed oil in garden cress. Similar study was conducted by Berehe and Boru [24] who suggested the crude extract from Ethiopian L. *sativum* seeds exhibited antimicrobial properties against tested fungi (*A. Niger*, *F. oxysporum*, and *F. solani*) and bacteria (*E. coli*, *S. typhi*, *B. subtilis*, and *S. aureus*). A study conducted by Adam et al. [25] also showed that petroleum ether, aqueous, and methanolic extracts of *L. sativum* seed obtained from Sudan exhibit antimicrobial activity against six opportunistic microorganisms: *S. aureus*, *E. coli*, *K. pneumoniae*, *Proteus vulgaris*, *P. aeruginosa*, and the fungus *C. albicans*. In this previous study, petroleum ether at different concentrations (2.5%, 5%, and 10%) was found to be a better solvent for extracting antimicrobial substances from *L. sativum* seeds than methanol and water.

## 5. Conclusion

The *Lepidium sativum* seed and leaf oil extracts were evaluated for physicochemical properties, antimicrobial, and antioxidant activities. *L. sativum* oil extracts were active against tested bacteria and fungus, suggesting their antimicrobial activity. The results revealed the antioxidant activities of seed oil were found to be higher than leaf oil extract. However, leaf oil has demonstrated better antimicrobial activities than seed oil. Garden cress seed can be used as a promising multipurpose medicinal source if further clinical trial is undertaken to prove its efficacy. The composition of the oil varies with harvesting or cultivation season, geographical location, genotypes of the crop plant, and extraction methods. It is difficult in the conditions of global drug resistance. Furthermore, the oil extract is nutritious and helps the animal host (human) to have both nutraceutical (development of the immune system, removal of free radicals like reactive oxygen species), as well as pharmaceutical (drug) properties. Hence, the oil extracts can be better preferred than antibiotic drugs. The antimicrobial component of the oil extracts may be due to the presence of various phenolic compounds and organic acids and bases in the oil depending upon the extraction conditions. Therefore, various extraction methods and oil processing methods should have to be tested for antimicrobial principles.

## Figures and Tables

**Table 1:** Physicochemical properties of garden cress seed and leaf oil extracts.

Oil extract	Oil yield (%)	Specific gravity	ACV (ml/g)	FFA (%)	PV (meqKOH/g)
Seed	25.75 ± 2.48a	0.84 ± 0.08a	1.26 ± 0.20b	0.63 ± 0.10b	2.20 ± 0.28b
Leaf	15.00 ± 0.71b	0.49 ± 0.02b	2.95 ± 0.20a	1.48 ± 0.09a	7.50 ± 0.71a

Note: means followed by the same letter within a column were not significantly different at the 0.05 probability level based on the LSD (least significance difference) test. Small letters: significance within column; ACV: acid value; FFA: free fatty acids; PV: peroxide value.

**Table 2:** Antioxidant activities of garden cress seed and leaf oil extracts.

Oil extract	DPPH (%)	HPSA (%)	AA (%)
Seed	26.55 ± 0.21a	32.35 ± 0.50b	24.21 ± 3.04a
Leaf	10.50 ± 0.71b	38.100 ± 0.42a	17.68 ± 0.25b

Note: means followed by the same letter within a column were not significantly different at the 0.05 probability level based on the LSD (least significance difference) test. Small letters: significance within column; DPPH: 2, 2- diphenyl-1-picrylhydrazyl; PSA: hydrogen peroxide scavenging activity; AA: ascorbic acid.

**Table 3:** Antimicrobial Activities oil extracts from garden cress seed and leaf oil extract as mean diameter of zone of inhibition against test pathogenic microbial spp.

Test pathogens	Oil extract	Concentrations of the oil extract (v/v)			Ciprofloxacin (1 *µ*g/ml)
		1*µ*g/ml	2*µ*g/ml	3*µ*g/ml	
*E. coli*	Seed	13.50 ± 0.50cC	15.87 ± 0.40bB	15.53 ± 0.45cB	18.33 ± 0.29aA
	Leaf	13.90 ± 0.36bcB	17.90 ± 0.25aA	17.50 ± 0.45bA	18.50 ± 0.50aA

*S. aureus*	Seed	14.57 ± 0.40bB	14.50 ± 0.32cB	17.23 ± 0.25bA	18.50 ± 0.50aA
	Leaf	15.50 ± 0.50aB	15.23 ± 0.36bB	18.50 ± 0.55aA	18.50 ± 0.45aA
					Fluconazole (1 *µ*g/ml)

*C. albicans*	Seed	9.00 ± 0.95bC	10.57 ± 0.40cC	12.57 ± 0.81bB	19.33 ± 0.76aA
	Leaf	13.50 ± 0.45aC	15.00 ± 0.50bB	18.00 ± 0.56aA	18.83 ± 0.76aA

*A. Niger*	Seed	7.73 ± 0.64bD	9.50 ± 0.50dC	12.87 ± 0.55bB	18.50 ± 0.76aA
	Leaf	13.50 ± 0.45aC	16.50 ± 0.60aB	18.50 ± 0.50aA	19.00 ± 0.46aA

Note: means followed by the same letter within a column were not significantly different at the 0.05 probability level based on LSD (least significance difference) *t* test. Small letters: significance within column; capital letters: significance across row. *E. coli*: *Escherichia coli*; *S. aureus*: *Staphylococcus aureus.*

**Table 4:** MIC, MBC, and MFC of garden cress seed and leaf oil extracts.

Test pathogens	Oil extract	MIC (*µ*g/ml)	MBC/MFC (*µ*g/ml)
*E. coli*	Seed	0.75	1.25
	Leaf	0.20	0.40

*S. aureus*	Seed	0.10	0.20
	Leaf	0.05	0.05

*A. Niger*	Seed	0.25	0.50
	Leaf	0.10	0.12

*C. albicans*	Seed	1.00	1.75
	Leaf	0.40	0.75

## Data Availability

The data used to support the findings of this study are included within supplementary information file.

## Supplementary Materials

Supplementary Materials The data used to support the findings of this study are included within supplementary information file.
