# Supplementary material for: Physicochemical Properties and Biological Activities of Garden Cress (Lepidium sativum L.) Seed and Leaf Oil Extracts
Source: Can J Infect Dis Med Microbiol. 2022 Mar 16;2022:2947836. doi: 10.1155/2022/2947836 (PMC8942675; doi:10.1155/2022/2947836)
Supplement: Supplementary Materials — The data used to support the findings of this study are included within supplementary information file. [file 2947836.f1.pdf]

Table 1. Data for physicochemical properties and antioxidant activities

Oil extract	rep	Oil yield	Spgr	Acv	FFA	PV	DPPH	PVsc	AA
seed	1	27.5	0.92	1.4	0.71	2	10	38.4	26.36
Seed	2	24	0.79	1.12	0.56	2.4	11	37.8	22.06
Leaf	1	15.5	0.51	2.81	1.41	8	26.7	32	17.5
Leaf	2	14.5	0.48	3.09	1.55	7	26.4	32.7	17.85

Spgr: specific gravity; ACV: acid value; FFA: free fatty acid value; PV: peroxide value; DPPH: 2, 2- diphenyl-1-picrylhydrazyl; PVSC: peroxide scavenging activity; AA: ascorbic acid.

Table 2. Data for antibacterial activity based on diameter of zone of inhibition

Test patho	Oil extract	rep	Concentrations of the oil extract (			Ciprofloxacin
			1µl/ml	2µl/ml	3µl/ml	(1µl/ml)
E. coli	Seed	1	13.5	15	16	18.5
E. coli	Seed	2	14	14.5	15.5	18.5
E. coli	Seed	3	13	14	15.1	18
E. coli	Leaf	1	14	15	17	19
E. coli	Leaf	2	13.5	15.5	18	18.5
E. coli	Leaf	3	14.2	15.2	17.5	18
S. aureus	Seed	1	15	15.5	17	18
S. aureus	Seed	2	14.5	16	17.5	18.5
S. aureus	Seed	3	14.2	16.1	17.2	19
S. aureus	Leaf	1	15	17.5	18	18.5
S. aureus	Leaf	2	16	18	18.5	18
S. aureus	Leaf	3	15.5	18.2	19	19

Table 3. Data for antifungal activity

Test patho	Oil extract	Rep	Concentration of the oil extract			Fulconazole
			1µl/ml	2µl/ml	3µl/ml	(1µl/ml)
C. albicans	Seed	1	9	10.5	12	20
C. albicans	Seed	2	10	11	13	18.5
C. albicans	Seed	3	8	10.2	13.6	19.5
C. albicans	Leaf	1	14	15.5	18	18
C. albicans	Leaf	2	13.5	15	18.5	19.5
C. albicans	Leaf	3	13	14.5	17.5	19
A. niger	seed	1	8	10	13	18
A. niger	seed	2	7	9	12	19.5
A. niger	seed	3	8.2	19.4	12.7	18
A. niger	Leaf	1	13	16	18	18.5
A. niger	Leaf	2	14	16.5	18.5	19.5
A. niger	leaf	3	13.5	17	19	19
